# Childhood Leukemia in Germany: Cluster Identified near Nuclear Power Plant

**Published:** 2007-06

**Authors:** Valerie J. Brown

Childhood leukemia clusters have been observed at a number of sites near European nuclear facilities. With the identification of the largest cluster to date, a new German study underscores the need to clarify the association **[*EHP* 115:947–952; Hoffmann et al.]**.

Between February 1990 and May 1991, five cases of leukemia were diagnosed in children living within 5 kilometers of the Krümmel nuclear power plant in Geesthacht and a neighboring nuclear research operation along the Elbe River in northern Germany. By 2005, another nine cases of leukemia had been discovered in the area. Most of the cases were acute lymphatic leukemia in males under five years of age.

Several expert commissions investigated, and found moderate levels of cesium in rain-water and air samples, along with plutonium and americium in household dust near the plant. There was also some evidence of chromosomal damage to lymphocytes among the local population. One panel deemed these observations consistent with fallout from a possible accident at the research facility that would have to have occurred around September 1986, but so far no such accident has been proved. Another panel suggested instead that chance or population mixing—the commingling of local people with newcomers from various places—might have caused the cluster.

In the current study, researchers compared the number of observed leukemia cases in the sparsely populated Geesthacht area to the number of predicted cases based on nearby county and national incidence rates from 1990 to 2005. The five cases found in 1990 and 1991 significantly exceeded the expected incidence for that period of 0.45 cases. After studying medical records from all treatment facilities in the vicinity and in Hamburg, the team concluded that the Geesthacht cluster is the “largest series of childhood leukemia cases reported to date” among European leukemia clusters near nuclear facilities, including those at Dounreay, Scotland; LeHague, France; and Sellafield, England.

The authors state that population mixing is unlikely to account for the leukemia incidence because the population remained stable over the years studied. Nor would an alleged one-time release of radiation in 1986 readily explain the cluster, given that the excess incidence persisted over at least 15 years. Thus, they conclude, the elevated incidence of childhood leukemia around Geesthacht remains “another piece in a growing puzzle” of childhood leukemia’s association with nuclear installations—and its severity and persistence emphasize the need to keep investigating.

## Figures and Tables

**Figure:**
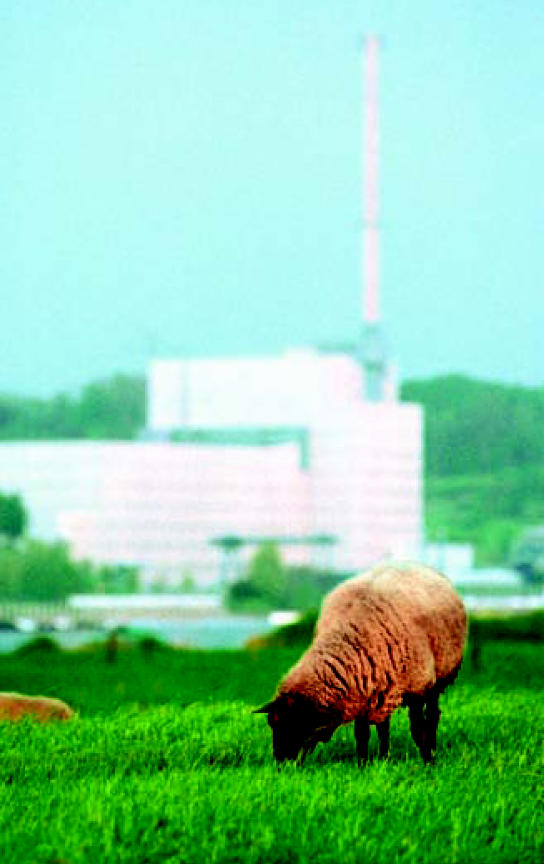
Krümmel nuclear power plant, Geestacht

